# Risk for Transportation of Coronavirus Disease from Wuhan to Other Cities in China

**DOI:** 10.3201/eid2605.200146

**Published:** 2020-05

**Authors:** Zhanwei Du, Lin Wang, Simon Cauchemez, Xiaoke Xu, Xianwen Wang, Benjamin J. Cowling, Lauren Ancel Meyers

**Affiliations:** University of Texas at Austin, Austin, Texas, USA (Z. Du, L.A. Meyers);; Institut Pasteur, Paris, France (L. Wang, S. Cauchemez);; Dalian Minzu University, Dalian, China (X. Xu);; Dalian University of Technology, Dalian (X. Wang);; The University of Hong Kong, Hong Kong (B.J. Cowling);; Santa Fe Institute, Santa Fe, New Mexico, USA (L.A. Meyers)

**Keywords:** Wuhan, coronavirus, COVID-19, epidemiology, importation, outbreak, viruses, China, 2019 novel coronavirus disease, severe acute respiratory syndrome coronavirus 2, SARS-CoV-2

## Abstract

On January 23, 2020, China quarantined Wuhan to contain coronavirus disease (COVID-19). We estimated the probability of transportation of COVID-19 from Wuhan to 369 other cities in China before the quarantine. Expected COVID-19 risk is >50% in 130 (95% CI 89–190) cities and >99% in the 4 largest metropolitan areas.

In December 2019, a novel coronavirus, since named severe acute respiratory syndrome coronavirus 2, emerged in Wuhan, China (*1*), causing a respiratory illness that the World Health Organization has named coronavirus disease (COVID-19). On January 30, 2020, the World Health Organization declared the outbreak a public health emergency of international concern (*2*). By January 31, 2020, a total of 192 fatalities and 3,215 laboratory-confirmed cases had been reported in Wuhan; 8,576 additional cases were spread across >300 cities in mainland China, and 127 exported cases were reported in 23 countries spanning Asia, Europe, Oceania, and North America. The rapid global expansion, rising fatalities, unknown animal reservoir, and evidence of person-to-person transmission potential (*3*,*4*) initially resembled the 2003 SARS epidemic and raised concerns about global spread.

On January 22, 2020, China announced a travel quarantine of Wuhan and by January 30 expanded the radius to include 16 cities, encompassing a population of 45 million. At the time of the quarantine, China was already 2 weeks into the 40-day Spring Festival, during which residents and visitors make several billion trips throughout China to celebrate the Lunar New Year (*5*). Considering the timing of exported COVID-2019 cases reported outside of China, we estimate that only 8.95% (95% credibility interval [CrI] 2.22%–28.72%) of persons infected in Wuhan by January 12 might have had COVID-19 confirmed by January 22. By limiting our estimate to infections occurring ≥10 days before the quarantine, we account for an ≈5–6-day incubation period and 4–5 days between symptom onset and case detection (Appendix) (*2*–*4*,*6*). The low detection rate coupled with an average lag of 10 days between infection and detection (*7*) suggest that newly infected persons who traveled out of Wuhan just before the quarantine might have remained infectious and undetected in dozens of cities in China for days to weeks. Moreover, these silent importations already might have seeded sustained outbreaks that were not immediately apparent.

We estimated the probability of transportation of infectious COVID-19 cases from Wuhan to cities throughout China before January 23 by using a simple model of exponential growth coupled with a stochastic model of human mobility among 369 cities in China (Appendix). Given that ≈98% of all trips taken during this period were made by train or car, our analysis of air, rail, and road travel data yields more granular risk estimates than possible with air passenger data alone (*8*).

By fitting our epidemiologic model to data on the first 19 cases reported outside of China, we estimate an epidemic doubling time of 7.31 days (95% CrI 6.26–9.66 days) and a cumulative total of 12,400 (95% CrI 3,112–58,465) infections in Wuhan by January 22 (Appendix). Both estimates are consistent with a similar epidemiologic analysis of the first 425 cases confirmed in Wuhan (*4*). Assuming these rates of early epidemic growth, we estimate that 130 cities in China have a >50% chance of having a COVID-19 case imported from Wuhan in the 3 weeks preceding the quarantine (Figure). By January 26, a total of 107 of these 130 high-risk cities had reported cases. However, 23 had not, including 5 cities with importation probabilities >99% and populations >2 million: Bazhong, Fushun, Laibin, Ziyang, and Chuxiong. 

**Figure:**
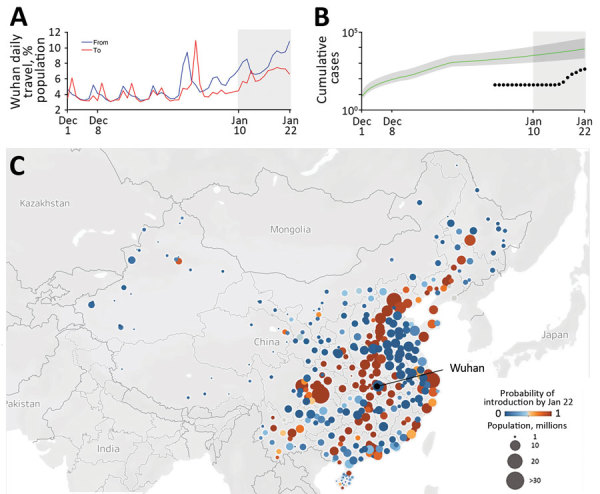
Risks for transportation of coronavirus disease (COVID-19) from Wuhan, China, before a quarantine was imposed on January 23, 2020. A) Daily travel volume to and from Wuhan, given as a percentage of the Wuhan population. Gray shading indicates the start of Spring Festival season on January 10, 2020, a peak travel period in China. B) Estimated and reported daily prevalence of COVID-19 in Wuhan. The green line and shading indicate model estimates of cumulative cases since December 1, 2019, with 95% credible interval bounds, assuming an epidemic doubling time of 7.31 days (95% credible interval 6.26–9.66 days). Black dots indicate cumulative confirmed case counts during January 1–22, 2020 (*10*). Gray shading at right indicates the start of Spring Festival season. C) Probability that >1 COVID-19 case infected in Wuhan traveled to cities in China by January 22, 2020. The 131 cities with a risk threshold >50% are indicated in shades of orange; 239 cities below that threshold are indicated in shades of blue. Map generated by using Mapbox (https://www.mapbox.com).

Under our lower bound estimate of 6.26 days for the doubling time, 190/369 cities lie above the 50% threshold for importation. Our risk assessment identified several cities throughout China likely to be harboring yet undetected cases of COVID-19 a week after the quarantine, suggesting that early 2020 ground and rail travel seeded cases far beyond the Wuhan region under quarantine.

Our conclusions are based on several key assumptions. To design our mobility model, we used data from Tencent (https://heat.qq.com), a major social media company that hosts applications including WeChat (≈1.13 billion active users in 2019) and QQ (≈808 million active users in 2019) (Statista, https://www.statista.com); consequently, our model might be demographically biased by the Tencent user base. Further, considerable uncertainty regarding the lag between infection and case detection remains. Our assumption of a 10-day lag is based on early estimates for the incubation period of COVID-19 (*4*) and prior estimates of the lag between symptom onset and detection for SARS (*9*). We expect that estimates for the doubling time and incidence of COVID-19 will improve as reconstructed linelists and more granular epidemiologic data become available (Appendix). However, our key qualitative insights likely are robust to these uncertainties, including extensive prequarantine exportations throughout China and far greater case counts in Wuhan than those reported before the quarantine.

Appendix Additional information on risk for transportation of 2019 novel coronavirus from Wuhan to other cities in China.
